# Time to treatment disparities in gastric cancer patients in the United States of America: a comprehensive retrospective analysis

**DOI:** 10.3389/fonc.2024.1292793

**Published:** 2024-02-09

**Authors:** Seema Sharan, Shivam Bansal, Harsheen Kaur Manaise, Paola Berrios Jimenez, Swathi R. Raikot, Syeda Hoorulain Ahmed, Reed Popp, Kyle Popp, Kulkaew Sukniam, Gabrielle Kowkabany, Fatima Mubarak, Emmanuel Gabriel

**Affiliations:** ^1^ Department of Surgery, Government Medical College and Hospital, Chandigarh, India; ^2^ Department of Surgery, University of Puerto Rico School of Medicine, San Juan, Puerto Rico; ^3^ Department of Surgery, Mayo Clinic, Rochester, MN, United States; ^4^ Department of Surgery, Dow University of Health Sciences, Karachi, Pakistan; ^5^ Department of Surgery, University of Florida College of Medicine, Gainesville, FL, United States; ^6^ Department of Surgery, Florida State University, Tallahassee, FL, United States; ^7^ Department of Surgery, Duke University Medical Center, Durham, NC, United States; ^8^ Department of Surgery, University of Alabama, Tuscaloosa, AL, United States; ^9^ Department of Surgery, Aga Khan University, Karachi, Pakistan; ^10^ Department of General Surgery, Mayo Clinic Florida, Jacksonville, FL, United States

**Keywords:** time to treatment, gastric cancer, disparities, disparities in treatment, cancer, sociodemographic factors

## Abstract

**Introduction:**

Gastric cancer ranks as the 5th most prevalent cancer and the 4th leading cause of cancer-related deaths worldwide. Various treatment modalities, including surgical resection, chemotherapy, and radiotherapy, are available for gastric cancer patients. However, disparities related to age, sex, race, socioeconomic factors, insurance status, and demographic factors often lead to delayed time to treatment.

**Methods:**

In this retrospective study, conducted between 2004 and 2019, we utilized data from the National Cancer Database (NCDB) to investigate the factors contributing to disparities in the time to first treatment, surgery, chemotherapy, and radiotherapy among gastric cancer patients. Our analysis incorporated several variables, and statistical analysis was conducted to provide valuable insights into these disparities.

**Results:**

We observed notable disparities in the timing of treatment for various demographic groups, including age, sex, race, insurance status, geographic location, and facility type. These disparities include longer time to treatment in males (32.67 vs 30.75), Native Americans (35.10 vs 31.09 in Asians), low-income patients (32 vs 31.15), patients getting treatment in an academic setting (36.11 vs 29.61 in community setting), significantly longer time to chemotherapy in 70+ age group (51.13 vs 40.38 in <40 y age group), black race (55.81 vs 47.05 in whites), low income people (49.64 vs 46.74), significantly longer time to radiotherapy in females (101.61 vs 79.75), blacks and Asians (109.68 and 113.96 respectively vs 92.68 in Native Americans) etc. There are various other disparities in time to surgery, chemotherapy, and radiotherapy.

**Conclusions:**

Understanding these disparities is crucial in developing targeted strategies to improve timely access to appropriate treatments and enhance outcomes for gastric cancer patients. Future research with updated data and prospective study designs can provide a more comprehensive understanding of the factors influencing patient outcomes in gastric cancer.

## 1 Introduction

In the year 2020, gastric cancer ranked as the 5th most prevalent cancer and the 4th leading cause of cancer-related deaths worldwide (1). Year 2020 reported over 1 million newly diagnosed cases of gastric cancer, with Eastern Asia and Eastern Europe reporting the highest incidences (2). In the United States, it is estimated that approximately 26,500 individuals will be diagnosed with gastric cancer in 2023, with the highest incidence observed in Japanese and Korean populations (2, 3). Despite a declining trend in incidence rates over the past few decades, the global burden of gastric cancer is projected to increase by 62% by 2040 (4). In the United States, black males and Hispanic females exhibit the highest incidence and mortality rates (5).

The primary causative agent of gastric cancer is Helicobacter pylori, responsible for nearly 90% of cases, while other risk factors include cigarette smoking, high salt diet, and processed meat consumption (2, 4). Various treatment modalities are available for gastric cancer, including surgical resection, chemotherapy, and radiotherapy. However, disparities related to age, sex, race, socioeconomic factors, insurance status, and demographic factors often lead to delayed time to treatment for patients with gastric cancer. While there are studies showing poor survival rates with a longer time to treatment in certain cancers (6), there is very little data demonstrating a correlation between time to treatment and overall survival in gastric cancer (5). For individuals who chose to undergo surgery as the initial treatment, there was a bimodal relationship concerning the time to treatment. Specifically, when the time to treatment was 8 weeks or less, a lengthier time to treatment correlated with an extended median overall survival. On the other hand, when the time to treatment ranged from 14 to 20 weeks, a prolonged time to treatment was linked to a diminished median overall survival (7).

This study aimed to investigate the different disparities affecting the time to treatment for individuals diagnosed with gastric cancer in the United States of America from 2004 to 2019. There have been studies done on disparities in gastric cancer treatment by Lemini et al. (8) and Rana et al. (9) but our paper focuses on more wider spectrum of sociodemographic groups including income, geographic location etc. as showed in below tables. By gaining a comprehensive understanding of these disparities, we may identify targeted strategies and interventions to improve timely access to appropriate treatments and enhance outcomes for all gastric cancer patients.

## 2 Methods

We performed a retrospective analysis utilizing data from the National Cancer Database (NCDB) covering the period from 2004 to 2019. The National Cancer Data Base (NCDB) is a comprehensive oncology outcomes database, capturing 70% of annual new invasive cancer diagnoses in the U.S. It serves as a crucial clinical surveillance and quality improvement tool for cancer programs under the American College of Surgeons Commission on Cancer approvals program. The information is employed to examine trends in cancer care, set benchmarks at regional and national levels, and facilitate quality improvement initiatives (9, 10). To access this data, the request was submitted through the American College of Surgeons to obtain the NCDB Participant User File (PUF), which is accessible to individuals affiliated with hospitals participating in the Commission on Cancer. Our study did not require Institutional Review Board approval. The study focused on patients who had been diagnosed with gastric cancer and adhered to the guidelines outlined by the American Joint Committee on Cancer (AJCC) 6th and 7th editions (11). The analysis encompassed a wide range of variables, including race, age, sex, income, insurance status, geographic location (rural/urban), treatment facility type, cancer stage, cancer grade, and Charlson-Deyo Comorbidity (CDC) score. Staging, grading and CDC scoring of gastric cancer is done similar to earlier studies (8, 11).

To evaluate the timing of treatment, specifically surgery, chemotherapy, and/or radiation, we computed and summarized the respective durations. Statistical analysis was performed using SAS version 9.4 (SAS Institute Inc., Cary, NC, USA).

Our research findings were presented through summaries of clinical and demographic characteristics, disease outcome measures, and treatment variables. For continuous variables, such as mean, median, standard deviation, and ranges, the Kruskal-Wallis test was utilized for analysis. Categorical variables were presented as frequencies and relative frequencies, and chi-square tests were employed for analysis.

By employing a robust methodology and rigorous statistical analysis, we aimed to provide valuable insights into the disparities related to the timing of treatment among gastric cancer patients and contribute to the advancement of knowledge in this field.

## 3 Results

### 3.1 Time to treatment

It is a crucial metric in cancer management, yet there is limited data comparing its impact on overall survival in patients with gastric cancer (5). Study by Ramanathan et al. indicates longer time to treatment has no correlation with overall survival (12) whereas according to Fisher et al. patients who had urgent surgery had worse outcomes as compared to elective surgery patients (13). Some studies indicate a bimodal relationship between overall survival and time to surgical treatment, with decreased survival rates observed in patients with treatment initiated either <4 weeks or >14 weeks after diagnosis (7). So, more studies are needed to know about the exact effect of time to treatment on overall survival. Notably, as shown in **Table 1**, longer time to treatment is associated with specific factors, including male sex, Native American race, Government Insurance, income less than 63000, and treatment received at academic settings.

**Table 1:** Time to first treatment.

		n	Mean (SD)	P-value
Age Categories	<40	6045	25.77 (33.85)	<.001
	40-50	14194	28.81 (33.76)	
	50-60	32153	31.47 (38.13)	
	60-70	45582	33.05 (37.60)	
	70+	68755	32.68 (37.07)	
Sex	Male	105474	**32.67** (36.09)	<.001
	Female	61255	30.75 (38.70)	
Race	White	126905	31.92 (35.66)	0.014
	Black	24877	32.30 (43.16)	
	Native American	643	**35.10** (39.06)	
	Asian	9052	31.09 (34.38)	
	Other	3572	32.44 (42.76)	
Rural/Urban	Metro	138964	31.87 (37.22)	0.47
	Urban	19753	32.21 (35.44)	
	Rural	2546	31.79 (39.21)	
Insurance Status	Not Insured	6007	28.88 (38.02)	<.001
	Private	59189	30.16 (34.87)	
	Government	97596	33.09 (38.16)	
	Unknown	3937	**35.96** (39.28)	
Income	<63,000	100688	**32.00 (**37.51)	<.001
	>63,000	54199	31.15 (35.63)	
Grade	Well	14185	32.50 (46.45)	<.001
	Moderately	37154	34.14 (35.04)	
	Poorly	77374	31.64 (31.91)	
	Undifferentiated	3226	26.96 (33.26)	
Stage	0	2110	34.94 (46.26)	<.001
	I	28159	38.72 (44.33)	
	II	20181	38.72 (33.60)	
	III	19570	34.84 (28.41)	
	IV	40865	29.06 (31.35)	
Facility Type	Community	13081	29.61 (33.85)	<.001
	Comprehensive	57737	28.83 (35.06)	
	Academic	68410	**36.11** (39.55)	
	Other	21456	30.39 (35.65)	

Bold values indicate longest time to treatment in the respective sociodemographic group.

### 3.2 Time to surgery

Surgery plays a pivotal role in the treatment of gastric cancer, with surgical intervention serving as the mainstay approach. For patients diagnosed with early-stage gastric cancer, total, subtotal, or distal gastrectomy with D2 lymphadenectomy offers a curative treatment option (14). In recent times, laparoscopic gastrectomy, performed by skilled surgeons, has gained prominence due to its associated benefits, such as reduced blood loss, fewer post-operative complications, and quicker recovery (15). A recent systematic review and meta-analysis of 16 studies comparing robotic gastrectomy (RG) and laparoscopic gastrectomy (LG) for gastric cancer indicated that RG is associated with a decreased risk of postoperative complications, shorter hospital stays, and lower rates of conversion to open surgery. Nevertheless, there were no significant differences observed in terms of overall survival, disease-free survival, or the number of harvested lymph nodes between the two procedures (16). However, disparities in time to surgery have been observed in certain patient groups as shown in **Table 2**. Males, Native Americans, individuals residing in urban areas, those with insurance coverage, and patients receiving treatment in academic settings experience longer time intervals before undergoing surgery.

**Table 2:** Time to surgery.

		n	Mean (SD)	P-value
Age Categories	<40	3415	55.75 (72.90)	<.001
	40-50	8784	60.61 (69.90)	
	50-60	20219	65.90 (73.30)	
	60-70	29750	65.54 (73.19)	
	70+	44497	47.67 (59.87)	
Sex	Male	64561	**63.05 (**69.25)	<.001
	Female	42104	48.82 (65.66)	
Race	White	79438	59.84 (67.35)	<.001
	Black	16651	49.39 (72.65)	
	Native American	393	**61.35** (77.63)	
	Asian	6718	47.88 (60.29)	
	Other	2327	59.73 (75.50)	
Rural/Urban	Metro	89020	56.59 (68.08)	<.001
	Urban	12387	**61.72 (**67.95)	
	Rural	1635	57.81 (68.14)	
Insurance Status	Not Insured	3148	51.76 (71.26)	<.001
	Private	38797	**63.37** (70.15)	
	Government	62699	54.20 (66.50)	
	Unknown	2021	52.78 (70.27)	
Income	<63,000	63671	55.67 (67.43)	<.001
	>63,000	34509	**57.78** (67.24)	
Grade	Well	12772	43.96 (63.09)	<.001
	Moderately	25356	62.04 (65.69)	
	Poorly	46747	60.87 (64.47)	
	Undifferentiated	2390	43.04 (59.10)	
Stage	0	2003	43.05 (62.93)	<.001
	I	24550	53.51 (61.74)	
	II	14997	93.61 (72.69)	
	III	12470	100.00 (73.08)	
	IV	5006	60.72 (91.28)	
Facility Type	Community	7511	42.49 (58.75)	<.001
	Comprehensive	35806	48.18 (61.48)	
	Academic	46025	**68.34 (**73.53)	
	Other	13908	53.67 (64.37)	

Bold values indicate longest time to treatment in the respective sociodemographic group.

### 3.3 Time to chemotherapy

It plays a vital role in the management of advanced gastric cancers, serving as a mainstay approach to improve patient outcomes. Studies have demonstrated that chemotherapy can extend survival by approximately 7 months compared to best supportive treatment (17). Additionally, neoadjuvant chemotherapy has been shown to reduce mortality in patients with advanced gastric cancer without increasing complications or post-operative mortality rates (18). However, certain patient groups experience longer time intervals before initiating chemotherapy as shown in **Table 3**. The 70+ age group, females, individuals of Asian or black race, those with low income, government insurance, and patients receiving treatment at academic settings tend to encounter delays in receiving chemotherapy.

**Table 3:** Time to chemotherapy.

		n	Mean (SD)	P-value
Age Categories	<40	4305	40.38 (38.36)	<.001
	40-50	9958	46.35 (44.54)	
	50-60	22055	47.71 (50.90)	
	60-70	29216	49.19 (50.85)	
	70+	32987	51.13 (45.07)	
Sex	Male	66950	47.76 (48.90)	<.001
	Female	31571	51.12 (45.72)	
Race	White	75848	47.05 (45.94)	<.001
	Black	14283	55.81 (57.17)	
	Native American	398	49.65 (38.74)	
	Asian	4876	55.77 (48.32)	
	Other	2216	49.33 (42.87)	
Rural/Urban	Metro	81592	48.95 (47.57)	0.22
	Urban	12148	48.14 (47.82)	
	Rural	1540	48.68 (41.50)	
Insurance Status	Not Insured	4304	48.58 (44.70)	<.001
	Private	39009	46.14 (48.40)	
	Government	52650	50.85 (47.67)	
	Unknown	2558	48.92 (49.41)	
Income	<63,000	59435	49.64 (48.54)	<.001
	>63,000	32126	46.74 (47.65)	
Grade	Well	3056	57.11 (79.18)	<.001
	Moderately	21260	50.51 (42.76)	
	Poorly	53054	49.20 (45.24)	
	Undifferentiated	1953	52.54 (43.84)	
Stage	0	197	71.63 (50.62)	<.001
	I	8753	64.42 (50.12)	
	II	15094	51.24 (38.88)	
	III	16451	45.61 (34.12)	
	IV	34471	36.00 (34.64)	
Facility Type	Community	7764	49.76 (45.49)	<.001
	Comprehensive	33873	47.01 (48.90)	
	Academic	40078	51.09 (46.58)	
	Other	12501	48.89 (53.21)	

### 3.4 Time to radiotherapy

It is a critical aspect in the management of locally advanced gastric cancer, particularly for alleviating local symptoms such as bleeding, pain, and obstruction. Palliative radiotherapy has shown promising response rates for these symptoms, with studies, like Tey et al. reporting rates as high as 74% for bleeding, 67% for pain, and 68% for obstruction (19). Low-dose radiotherapy is often preferred to minimize adverse effects, as it yields similar response rates with fewer side effects (19). However, certain patient groups experience delays in receiving radiotherapy as shown in **Table 4**. Females, individuals of black and Asian ethnicity, uninsured patients, and those residing in metropolitan cities tend to encounter longer intervals before commencing radiotherapy.

**Table 4:** Time to radiotherapy.

		n	Mean (SD)	P-value
Age Categories	<40	946	97.62 (73.49)	<.001
	40-50	2836	92.11 (99.44)	
	50-60	6800	84.97 (65.95)	
	60-70	9132	81.67 (63.51)	
	70+	7938	87.42 (60.02)	
Sex	Male	20059	79.75 (62.04)	<.001
	Female	7593	**101.61** (80.11)	
Race	White	21798	79.41 (67.40)	<.001
	Black	3433	109.68 (66.14)	
	Native American	91	92.68 (71.39)	
	Asian	1514	**113.96** (61.00)	
	Other	591	105.89 (72.93)	
Rural/Urban	Metro	22576	**87.95 (**69.66)	<.001
	Urban	3682	75.58 (61.17)	
	Rural	471	75.08 (53.56)	
Insurance Status	Not Insured	968	**99.00** (71.56)	<.001
	Private	12311	82.30 (63.56)	
	Government	13919	87.55 (63.80)	
	Unknown	454	96.04 (192.69)	
Income	<63,000	16476	86.48 (70.88)	0.08
	>63,000	8964	84.90 (63.50)	
Grade	Well	897	79.44 (66.21)	<.001
	Moderately	7396	76.66 (59.11)	
	Poorly	16363	92.04 (65.24)	
	Undifferentiated	547	94.48 (62.23)	
Stage	0	90	107.26 (64.00)	<.001
	I	3166	99.92 (67.08)	
	II	6173	77.72 (65.53)	
	III	6973	65.57 (56.55)	
	IV	1303	79.14 (82.18)	
Facility Type	Community	2028	88.88 (61.90)	0.052
	Comprehensive	9447	85.55 (74.07)	
	Academic	11305	85.02 (65.52)	
	Other	3926	83.85 (62.16)	

Bold values indicate longest time to treatment in the respective sociodemographic group.

## 4 Discussion

In our study, we have investigated the factors contributing to disparities in the time to first treatment, surgery, chemotherapy, and radiotherapy among gastric cancer patients. Age, sex, race, insurance, income, facility type, and geographic setting emerged as influential factors influencing these treatment timelines.

Intriguingly, our data reveals that the time to surgery is notably shorter for individuals aged <40 years and >70 years compared to other age groups. The primary reason for this disparity likely lies in the early-stage diagnosis and lower prevalence of comorbidities among individuals under 40 years, making them highly suitable candidates for early surgical intervention (20). Conversely, patients over 70 years of age are typically diagnosed at an advanced stage of the disease, necessitating immediate surgical action due to the severity of their condition. This finding aligns with the observations made by Brenkman et al., who demonstrated that individuals with advanced tumor stages undergo surgery more promptly than those with early-stage tumors (21).

In our study, a notable disparity was observed between females and males regarding the time to first treatment and surgery, as well as the time to chemotherapy and radiotherapy. The findings suggest that females tended to receive earlier treatment, particularly surgical intervention, potentially hindering the progression to advanced stages of gastric cancer. In contrast, males experienced delays in surgical treatment compared to females, leading to a higher incidence of advanced-stage disease and subsequent initiation of chemotherapy and radiotherapy at an earlier stage.

The underlying reasons for this sex-based variation may stem from inherent sex-related characteristics. Males, in general, have been reported to display reluctance in utilizing healthcare services, whereas females tend to be more frequent users of such services (22). This discrepancy in healthcare-seeking behavior may contribute to the observed differences in treatment timelines. Furthermore, existing literature on other cancer types, such as lung cancer, has shown that women are more likely than men to opt for surgical treatments. This factor may account for the prevention of advanced disease progression in females, leaving males with limited options and necessitating earlier reliance on chemotherapy and radiotherapy (22).

In the studied population, we observed significant differences in the time to first treatment and time to surgery between the Asian and Native American groups. Asian individuals exhibited notably shorter intervals to treatment initiation and surgical intervention, while Native Americans experienced prolonged time to treatment and surgery, yet shorter time to chemotherapy and radiotherapy. This disparity can be attributed to higher awareness levels among Asian individuals regarding gastric cancer, likely influenced by the higher incidence of gastric cancer in this population. Consequently, this heightened awareness leads to earlier diagnosis, rendering Asian individuals better candidates for prompt surgical treatment (5, 8, 23, 24).

It is a known fact that gastric cancer patients without insurance have higher mortality rates compared to those with insurance (9, 25). Surprisingly, patients without insurance demonstrated significantly shorter time to treatment and time to surgery when compared to their insured counterparts. This observation may seem counterintuitive at first, but the most plausible explanation for this difference lies in the worse presentation of the disease at the time of diagnosis among patients without insurance, which necessitates more urgent surgical intervention.

Patients without insurance often face barriers to accessing healthcare services, resulting in delayed diagnosis and limited access to regular medical care. Consequently, gastric cancer may be detected at more advanced stages, leading to a more critical condition that requires immediate surgical intervention. On the other hand, patients with insurance, who likely have better access to healthcare services and early diagnosis, may have the luxury of time for a more comprehensive evaluation and preparatory measures before surgery.

Our study unveiled a concerning trend among gastric cancer patients, indicating that individuals with lower income experience significantly longer intervals to treatment, chemotherapy, and radiotherapy. Notably, this disparity may be compounded by lower education levels, which further diminish the likelihood of receiving timely medical intervention, possibly due to limited health literacy and a lack of awareness regarding the potential consequences of forgoing treatment (23, 26, 27). Tragically, this phenomenon contributes to higher mortality rates among gastric cancer patients from lower-income backgrounds and with lower educational attainment (28).

In our study, we observed notable differences in the time to treatment, surgery, and chemotherapy between patients receiving care in academic settings and those in other healthcare settings. This observation is consistent with findings from previous research for gastric cancer patients and patients with other malignancies (6, 29). The longer treatment duration at academic centers could be attributed to several factors, including a higher patient load, reduced scheduling flexibility, and a substantial number of referral cases that necessitate thorough reanalysis before offering treatment. Academic medical centers often serve as referral centers, receiving complex cases from various regions. Consequently, the need for comprehensive evaluations and consultations can lead to longer timeframes before treatment initiation.

Similarly, time to surgery and radiotherapy was more prolonged in patients living in urban and metropolitan cities respectively than in rural areas. Clinics in rural areas often experience a lower patient load than their urban and metropolitan counterparts. This lower patient volume can be advantageous in offering early appointments and enabling timely treatment for patients in rural communities.

It is essential to acknowledge that while the study identified statistically significant disparities in various factors, these differences may not have significant clinical implications. The large number of patients included in the database enabled the attainment of statistical significance, but the actual differences in the time to treatment among various treatment variables might not result in substantial variations in patient outcomes. While our study extensively covers various aspects in time to treatment disparities of gastric cancer, the analysis of survival rates is beyond the scope of our study. Future research could explore survival rates in the context of gastric cancer.

Although utilizing a large database enhances the generalizability of the study, it is crucial to recognize it as a noteworthy limitation. The sheer volume of patient information may lead to missing data and inaccurately documented information, potentially affecting the study’s reliability. Additionally, the study’s retrospective nature poses limitations, as the data might not fully represent current practices and disparities in the field. Changes in healthcare practices and advancements in treatments over time could influence the relevance of the study’s findings to current medical practices. Moreover, the inherent variability in hospital charges for cancer treatment poses a limiting factor. Different hospitals, particularly private institutions versus safety net hospitals, may employ distinct cost structures for healthcare services which could result in different time to treatment for patients. NCDB, which serves as the primary data source for our study does not provide any information on this. Nonetheless, our study appears to be the first that analyses disparities among gastric cancer patients across all stages and socioeconomic factors.

## 5 Conclusions

In conclusion, our retrospective study identified significant disparities in the time to treatment, surgery, chemotherapy, and radiotherapy among gastric cancer patients. Age, sex, race, insurance, income, facility type, and geographic setting were key factors influencing treatment timelines. Understanding these disparities is crucial for targeted interventions to improve timely access to care and enhance patient outcomes. However, it is important to interpret the findings cautiously due to the potential limitations of the study. Future research with updated data and prospective designs will further enhance our understanding of these disparities and help develop effective strategies to address them.

## Data availability statement

The original contributions presented in the study are included in the article/supplementary material. Further inquiries can be directed to the corresponding author.

## Author contributions

SS: Writing – original draft, Writing – review & editing. SB: Writing – original draft, Writing – review & editing. EG: Supervision, Writing – review & editing. HM: Writing – review & editing. PJ: Writing – review & editing. SR: Writing – review & editing. SA: Writing – review & editing. RP: Writing – review & editing. KP: Writing – review & editing. KS: Writing – review & editing. GK: Writing – review & editing. FM: Writing – review & editing.
